# The Role of [^18^F]FDG PET/CT in Predicting Toxicity in Patients with NHL Treated with CAR-T: A Systematic Review

**DOI:** 10.3390/tomography10060066

**Published:** 2024-06-03

**Authors:** Natale Quartuccio, Salvatore Ialuna, Sabina Pulizzi, Dante D’Oppido, Stefania Nicolosi, Antonino Maria Moreci

**Affiliations:** Nuclear Medicine Unit, Ospedali Riuniti Villa Sofia—Cervello, 90146 Palermo, Italy; n.quartuccio@villasofia.it (N.Q.);

**Keywords:** positron emission tomography, [^18^F]FDG, lymphoma, NHL, CAR-T, toxicity

## Abstract

CAR-T-cell therapy, also referred to as chimeric antigen receptor T-cell therapy, is a novel method in the field of immunotherapy for the treatment of non-Hodgkin’s lymphoma (NHL). In patients receiving CAR-T-cell therapy, fluorodeoxyglucose Positron Emission Tomography/Computer Tomography ([^18^F]FDG PET/CT) plays a critical role in tracking treatment response and evaluating the immunotherapy’s overall efficacy. The aim of this study is to provide a systematic review of the literature on the studies aiming to assess and predict toxicity by means of [^18^F]FDG PET/CT in patients with NHL receiving CAR-T-cell therapy. PubMed/MEDLINE and Cochrane Central Register of Controlled Trials (CENTRAL) databases were interrogated by two investigators to seek studies involving the use of [^18^F]FDG PET/CT in patients with lymphoma undergoing CAR-T-cell therapy. The comprehensive computer literature search allowed 11 studies to be included. The risk of bias for the studies included in the systematic review was scored as low by using version 2 of the “Quality Assessment of Diagnostic Accuracy Studies” tool (QUADAS-2). The current literature emphasizes the role of [^18^F]FDG PET/CT in assessing and predicting toxicity in patients with NHL receiving CAR-T-cell therapy, highlighting the evolving nature of research in CAR-T-cell therapy. Additional studies are warranted to increase the collected evidence in the literature.

## 1. Introduction

Non-Hodgkin’s lymphoma (NHL) is the most common type of blood cancer worldwide, encompassing a variety of B- and T-cell proliferations. It is distinguished from Hodgkin’s lymphoma by its distinctive clinical symptoms and histological characteristics [1]. Approximately 260,000 deaths and 545,000 new cases were linked to non-Hodgkin’s lymphoma (NHL) worldwide in 2020. In contrast to highly developed countries, North African countries had a somewhat greater death burden, with Australia and New Zealand exhibiting the most apparent rising trend. The older population has shown the largest Average Annual Percent Change (AAPC) at 4.9 (95%CI: 3.6–6.2) and 6.8 (95%CI: 4.3–9.2), respectively, as the rates of growth in both incidence and death have accelerated over the previous few decades. It is predicted that by 2040, there will be roughly 778,000 NHL incident cases due to changes in demographics [2].

CAR-T-cell therapy, also referred to as chimeric antigen receptor T-cell therapy, is a novel method in the field of immunotherapy for the treatment of cancer. Reprogramming a patient’s own T cells to express a synthetic receptor known as a chimeric antigen receptor (CAR), which selectively targets cancer cells, is the novel therapeutic approach [3]. The first step in the procedure is to remove the patient’s T cells, which are immune cells, and then genetically alter them to manufacture CARs on their surface. After being grown in a lab, these modified CAR-T cells are subsequently reinfused into the patient [4]. After entering the body, CAR-T cells may identify and attach to particular proteins on the surface of cancer cells, which causes the cells to be destroyed. CAR-T-cell treatment has been shown to be effective in treating leukemia and lymphoma and gives people who have tried every other kind of treatment hope again [4].

In patients receiving CAR-T-cell therapy, fluorodeoxyglucose Positron Emission Tomography/Computer Tomography ([^18^F]FDG PET/CT) plays a critical role in tracking treatment response and evaluating the immunotherapy’s overall efficacy. [^18^F]FDG PET/CT is a potent imaging technique that makes it possible to visualize metabolic activity within the body, especially in quickly proliferating cells like cancer cells. [^18^F]FDG PET/CT scans are important tools in the CAR-T-cell therapy environment for identifying and localizing recurrent or residual disease, assessing the degree of treatment response, and assisting doctors in making well-informed decisions for the continued care of their patients. By tracking the metabolic alterations using [^18^F]FDG PET/CT scans, important details regarding the survival or removal of cancerous cells can be obtained, which helps in the early detection of recurrence. Thus, this imaging method adds to a comprehensive strategy for post-CAR-T-cell therapy patient management, guaranteeing prompt intervention and individualized care based on each patient’s unique response to treatment.

As research into this revolutionary therapy progresses, there are still obstacles to overcome, such as controlling possible adverse effects and extending its use to solid tumors [5]. A significant concern is the potential toxicity associated with CAR-T-cell therapy. Although CAR-T cells have demonstrated impressive results in the treatment of some cancers by using the patient’s immune system to identify and eliminate cancer cells, their strong activity may have unfavorable consequences. Cytokine release syndrome (CRS), a systemic inflammatory reaction brought on by the quick release of cytokines from activated CAR-T cells, is one frequent hazard. From fever and flu-like symptoms to more serious consequences like organ malfunction, CRS can present with a variety of symptoms. Another noteworthy issue is neurotoxicity, which is typified by neurological symptoms like convulsions and confusion. In order to guarantee the general safety and effectiveness of CAR-T-cell therapy, efforts are being made to reduce these toxicities through the creation of novel CAR-T cell designs, such as those with programmable switches and suicide genes, as well as through the improvement of patient care techniques. To further improve therapeutic outcomes and expand the use of this potential cancer treatment, ongoing research and clinical studies are being conducted to gain a deeper understanding of the toxicity of CAR-T cells [6]. [^18^F] FDG PET/CT may serve as a valuable tool in patients with lymphoma receiving CAR-T-cell therapy because it can track alterations in the body’s metabolism, particularly those connected to inflammation and tumor response, which can be signs of possible toxicities such as CRS and neurotoxicity.

The aim of this study is to provide a systematic review of the literature on the studies using [^18^F]FDG PET/CT for assessing toxicity and aiming to predict toxicity in patients with NHL receiving CAR-T-cell therapy.

## 2. Materials and Methods

The systematic review was conducted in accordance with the Preferred Reporting Items for Systematic Reviews and Meta-Analyses (PRISMA) 2020 checklist [7]. Before starting the literature search, a protocol was developed defining the research question, search methods, inclusion criteria, quality assessment, data extraction and statistical analysis.

### 2.1. Literature Search

PubMed/MEDLINE and Cochrane Central Register of Controlled Trials (CENTRAL) databases were interrogated by two investigators to seek studies involving the use of [^18^F]FDG PET/CT in patients with lymphoma undergoing CAR-T cell therapy. The literature search was launched on 9 January 2024, Bethesda, time: 12 pm, for both databases. No language restriction or start period was applied.

The search string for the literature search in PubMed/MEDLINE was: (“Positron Emission Tomography Computed Tomography”[Mesh] OR PET OR PET/CT) AND (“Lymphoma”[Mesh] OR DLBCL) AND (“Fluorodeoxyglucose F18”[Mesh] OR FDG OR 18F-FDG) AND (“axicabtagene ciloleucel” [Supplementary Concept] OR “tisagenlecleucel” [Supplementary Concept] OR “brexucabtagene autoleucel” [Supplementary Concept] OR “idecabtagene vicleucel” [Supplementary Concept] OR CAR-T OR “antigen receptor T-cell therapy” OR “Chimeric antigen receptor T-cell”).

The string used for the search in CENTRAL was (Positron Emission Tomography Computed Tomography OR PET OR PET/CT) AND (Lymphoma OR DLBCL) AND (Fluorodeoxyglucose OR FDG OR 18F-FDG) AND (axicabtagene ciloleucel OR tisagenlecleucel OR brexucabtagene autoleucel OR idecabtagene vicleucel OR CAR-T OR antigen receptor T-cell therapy OR Chimeric antigen receptor T-cell).

The literature search was updated until 2 May 2024, Bethesda, time: 12 pm, for both databases.

### 2.2. Study Selection

All identified references were exported to a reference management software (Endnote v. X7.5, Clarivate Analytics Philadelphia, PA, USA). A researcher screened the titles and abstracts of the retrieved entries to exclude duplicated articles, articles out of the topic of the present study, or non-original articles. The full text of the remaining articles was retrieved to verify the following inclusion criteria: (1) a study cohort or a subset of a minimum of 10 patients with lymphoma undergoing CAR-T-cell therapy and [^18^F]FDG PET/CT; (2) no evidence of other malignancies in patient history. A formal request was forwarded via email to the corresponding author in case of an unavailable full-text for download. The references of the selected articles were also screened for additional studies.

### 2.3. Data Extraction

Two researchers independently extracted data from all included studies and any disagreement was resolved in a consensus meeting. Bibliographical and technical data were extracted from the articles for inclusion in a descriptive table.

### 2.4. Methodological Quality Assessment

The methodological quality of the studies was assessed by an investigator using version 2 of the “Quality Assessment of Diagnostic Accuracy Studies” tool (QUADAS-2) [8], which comprises four domains: patient selection, index test, reference standard, flow and timing. The concerns about the risk of bias or applicability were described as low, high or unclear.

## 3. Results

### 3.1. Literature Search and Eligibility Assessment

**Figure 1:**
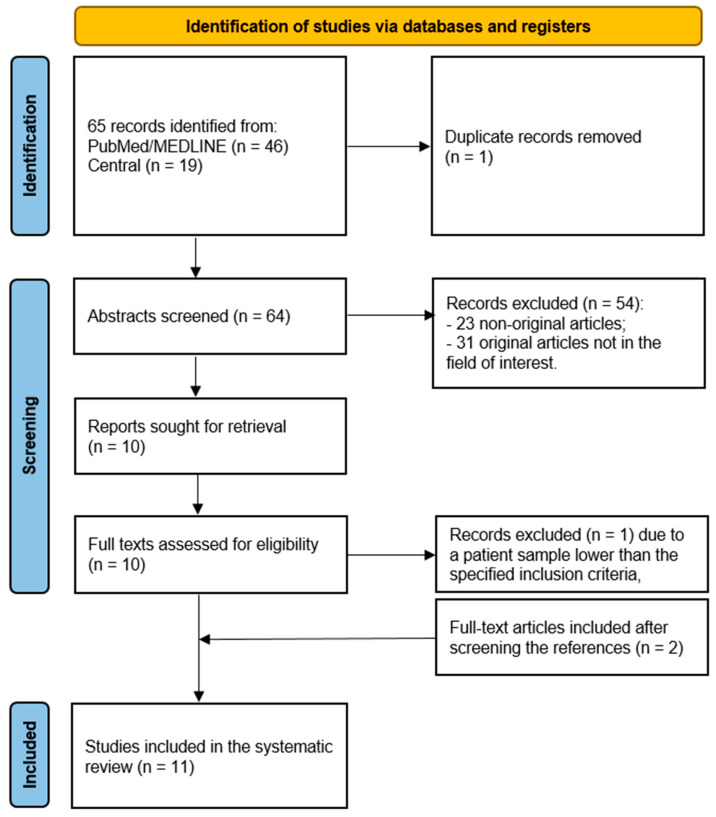
Flowchart of the literature search.

The comprehensive computer literature search revealed 65 articles (Figure 1). After importing the articles in the reference manager, one article was removed because it was a duplicate. Reviewing titles and abstracts, a total of 54 entries were excluded because they were non-original articles (*n* = 23) or were not in the field of interest of the systematic review (*n* = 31). The full text of the remaining 10 studies was retrieved and evaluated to check the inclusion criteria. After checking the full text, one article was excluded due to a patient number (*n* = 6) lower than the specified inclusion criteria. Two additional records were retrieved and included in the systematic review after crosschecking the references, leading to a final selection of 11 original studies. The main characteristics of the 11 studies with a total number of 833 patients included in the systematic review are presented in Table 1.

### 3.2. Methodological Quality of Included Studies

The risk of bias for the studies included in the systematic review was scored as low by using the QUADAS-2 for most of the studies (Table 2).

### 3.3. Systematic Review

It is still unknown how systemic inflammation, lymphoid organ function, and lymphoma activity relate to one another in patients treated with CD19-targeting CAR-T-cell immunotherapy and what that means for treatment response and side effects. Derlin et al. analyzed ten patients receiving treatment for relapsed or refractory diffuse large B-cell lymphoma with Tisagenlecleucel, an autologous CD19 CAR-T-cell product, using serial [^18^F]FDG PET/CT scans. Both lymphoma and lymphoid organ metabolic characteristics were evaluated, and the frequency and degree of toxicity (particularly neurotoxicity) were noted. Interestingly, four patients developed neurotoxicity. While the total lesion glycolysis (TLG) (*p* = 0.1099) and metabolic tumor volume (MTV) (*p* = 0.1041) did not show a significant difference (33.2  ±  8.8, range 26.9–46.3, compared to 22.3  ±  6.2, range 12.2–28.4; *p* = 0.0489), the maximum standardized uptake value (SUVmax) at baseline was noticeably higher in patients developing neurotoxicity compared to patients without neurotoxicity [33.2  ±  8.8 (range 26.9–46.3) vs. 22.3  ±  6.2 (range 12.2–28.4), respectively; (*p* = 0.0489)]. Furthermore, CRS occurred in four patients (40%), but no baseline PET1 parameter was significantly associated with the development of CRS (*p*  ≥  0.0822 in all cases). An early metabolic response was revealed to be necessary for remission (*p* = 0.0476). On the other hand, a less favorable result was linked to an early drop in metabolic activity in lymphoid organs such as the lymph nodes (*p* = 0.0470) and spleen (*p* = 0.0368) [8]. Another study (multicenter and retrospective), including 329 patients with large B-cell lymphoma (LBCL) who received commercial anti-CD19 CAR-T-cell therapy, aimed to evaluate the prognostic implications of early metabolic response on long-term outcomes. Elevated baseline lactate dehydrogenase, grade 3 or higher cytokine release syndrome, and Deauville scores (DS) of 4 or 5 on the one-month PET/CT were associated with a higher risk of progression and toxicity. These indicators suggest that PET/CT scans can provide critical information about potential complications and the likelihood of adverse events [13].

PET/CT also demonstrates the ability to predict the risk of toxicity, and its parameters may correlate with the severity of toxicity. The study by Hong et al. examined the relationship between PET/CT outcomes and toxicity in 41 patients treated with CAR-T-cell therapy for relapsed or refractory non-Hodgkin lymphoma (R/R NHL). Patients with higher baseline values of MTV, TLG, and average maximum standardized uptake value (SUVavg) were at increased risk of severe CRS following therapy. Baseline SUVavg was an independent risk factor for CRS, indicating that patients with a higher baseline tumor metabolic burden were more likely to experience severe CRS. Baseline TLG was found to be strongly correlated with peak serum cytokine levels during CRS incidence, including IL-6, IFN-γ, ferritin, and D-dimer [18]. These findings underscore the importance of PET/CT imaging in identifying and assessing toxicity risks, such as coagulation issues and CRS, in patients undergoing CAR-T-cell therapy.

Identifying high-risk patients can also guide clinical decision-making and personalized treatment plans. In a study, Gui et al. involved 38 patients with diffuse large B-cell lymphoma (DLBCL) who received CAR-T-cell therapy, and found that PET/CT metabolic parameters (SUVmax, TLG, and changes in these parameters), played a key role in predicting patient outcomes and toxicity following therapy. In particular, SUVmax and TLG before CAR-T-cell infusion were strongly correlated with the severity and risk of CRS [9]. The study by Leithner et al., looked at the relationship between PET/CT findings and toxicity in 180 patients with LBCL receiving autologous CD19-directed CAR-T treatment. PET/CT scans were examined for a variety of metrics, including SUVmax, MTV, TLG, and radiomic features. Higher CAR-PET MTV was linked to an increased risk of CRS.

Other authors evaluated both the predictive and prognostic capabilities of [^18^F]FDG PET. The purpose of the study by Marchal and colleagues was to find [^18^F]FDG PET biomarkers predictive of adverse events and related to prognosis in patients receiving CAR-T-cell treatment [9]. Patients treated with CAR-T cells were retrieved retrospectively from the databases of two university hospitals. Just before the infusion of CAR-T cells, [^18^F]FDG PET scans were conducted, and lesions were semi-automatically segmented using a threshold of 41% of the maximal uptake. The following data were gathered: SUVmax, total metabolic tumor volume (TMTV), uptake intensity of the liver and healthy lymphoid organs, and sDmax (a new feature that defines the distance between the two farthest lesions on the body surface, measured and standardized for accuracy). Progression-free survival (PFS) and overall survival (OS) were estimated using the Kaplan-Meier technique. Adverse effects such as immune effector cell-associated neurotoxicity syndrome (ICANS) and CRS were recorded. There were fifty-six patients in total, and their median follow-up was 9.7 months. Using multivariate analysis, it was found that sDmax (cut-off of 0.15 m^−1^) independently predicted OS (*p* = 0.008) and that TMTV (cut-off of 36 mL) was an independent predictive factor for PFS (*p* < 0.001). Regarding side effects, before CAR-T-cell infusion, higher levels of C-reactive protein (>35 mg/L, *p* = 0.006) and liver SUVmean (>2.5, *p* = 0.027) were connected to grade 2 to 4 CRS, while higher levels of spleen SUVmean (>1.9) were connected to grade 2 to 4 ICANS. In the study by Ababneh et al., reduced pre-CAR-T TLG and MTV were linked to better OS and complete response rates in 59 patients undergoing CAR-T-cell therapy. Significant correlations were found between high TLG and any-grade CRS and between developing any-grade ICANS events and high MTV. Significant correlations were found between high SUVmax and grade 3–4 neurological episodes. While high TLG pre-CAR-T was found to be a major prognostic factor for worse PFS, high MTV post-CAR-T was found to be the most important prognostic factor for shorter OS. In addition, shorter OS was linked to greater MTV, TLG, and SUVmax post-CAR-T.

Another group of researchers focused on brain [^18^F]FDG PET findings in patients receiving CAR-T therapy. In the study by Morbelli et al. [10], before and 30 days after starting CAR-T therapy, 21 patients with resistant diffuse large B-cell lymphomas (DLCBLs) underwent whole-body and brain [^18^F]FDG PET scans. Of them, five did not have any inflammatory side effects; eleven had CRS, and five of them progressed from CRS to ICANS. Brain [^18^F]FDG PET scans obtained before and after CAR-T treatment were compared with a local control dataset to detect hypometabolic trends at the patient and group levels (*p* < 0.05 after family wise error [FWE] correction). Using baseline [^18^F]FDG PET, MTV and TLG were computed, and a *t*-test was used to compare the results across patient subgroups. ICANS revealed a bilateral hypometabolic pattern that was extensive and mostly affected the anterior cingulate, frontal dorsolateral cortex, and orbitofrontal cortex (*p* < 0.003 FWE-corrected). In less extensive clusters, CRS without ICANS showed significant hypometabolism (*p* < 0.002 FWE-corrected), mainly in the bilateral medial and lateral temporal lobes, posterior parietal lobes, anterior cingulate, and cerebellum. In contrast to CRS, ICANS showed more noticeable hypometabolism in the frontal dorsolateral cortex and orbitofrontal cortex in both hemispheres (*p* < 0.002 FWE-corrected). In ICANS compared to CRS, the mean baseline MTV and TLG were considerably greater (*p* < 0.02).

Targeting CD19, CAR-T-cell therapy has shown great efficacy in treating patients with relapsed or resistant non-Hodgkin lymphoma (NHL). Nevertheless, it has been associated with notable side effects, most notably CRS. Though a thorough investigation is missing, prior research has conjectured about the influence of NHL baseline disease burden on both clinical outcomes and CRS. In the study by Wang et al. [11], for 19 NHL patients receiving CAR-T-cell therapy, the authors measured MTV and TLG using [^18^F]FDG PET/CT as quantitative indices of baseline tumor burden. The median MTV and TLG for the pre- and post-CAR-T-cell therapy were 72 cm^3^ (range: 0.02 to 1137.7 cm^3^) and 555.9 (range: 0.011 to 8990.3), respectively. The best overall response rate after a follow-up of a median of five months (range, one to twelve months) was 79.0%. Notably, responders and non-responders did not vary significantly in their baseline MTV or TLG (*p* = 0.62 and 0.95, respectively). Baseline MTV and TLG did not significantly correlate with overall survival, according to Cox regression analysis (*p* = 0.67 and 0.45, respectively). In contrast to patients with severe CRS (grades 3 to 4), individuals with mild to moderate CRS (grades 0 to 2) showed considerably lower MTV and TLG (*p* = 0.008 for MTV comparison, *p* = 0.011 for TLG comparison). Additionally, pseudoprogression and local immune activation linked to CAR-T-cell therapy in NHL patients were revealed by [^18^F]FDG PET/CT. These results highlight the frequency of lymphoma pseudoprogression and local immune activation during CAR-T-cell therapy and point to a connection between higher baseline disease load and more severe CRS.

CAR-T-cell treatment can be administered to outpatients, but it requires close observation for possible adverse effects, including CRS and ICANS. Although pre-infusion tumor burden and CRS are associated, information about the importance of pre-infusion tumor growth rate (TGR) is not currently available. The goal of the study by Winkelmann and colleagues was to evaluate the effect of TGR on the incidence and seriousness of ICANS and CRS. Prior to CAR-T injection, they included consecutive patients with pre-baseline and baseline (BL) imaging. Over the days between exams, TGR was computed as the absolute (abs) and percentage change (%) of the tumor burden based on the Lugano criteria. The consensus criteria of the American Society for Transplantation and Cellular Therapy (ASTCT) were used to grade CRS and ICANS. Clinical information was gathered, including patient age, ECOG (Eastern Cooperative Oncology Group) performance status, LDH (lactate dehydrogenase), and the international prognostic index (IPI). There were sixty-two patients (median age, 62 years, 40% female). Pre-BL TGR [%] and [abs] had median values of 30.9%/d and 7.5 mm^2^/d, respectively. Pre-BL TGR [abs] and pre-BL TGR [%] showed no connection with ICANS (r[abs] = −0.06 and r[%] = −0.07) and a very minor positive correlation with the grade of CRS (r[abs] = 0.14 and r[%] = 0.13). While no significant association was found between CRS or ICANS and the other parameters that were evaluated, there was a weak positive link between CRS grade and ICANS grade (r = 0.35; *p* = 0.005). Prior to CAR-T, pre-infusion TGR did not significantly predict ICANS and only weakly correlated with the occurrence of CRS, not its severity. Crucially, compared to pre-infusion tumor burden alone, pre-infusion TGR did not yield any new information. Therefore, pre-infusion TGR should not have an impact on outpatient planning or toxicity management [12].

A summary of the main findings and types of toxicity documented in the studies included in the present systematic review is presented in Table 3.

## 4. Discussion

Our systematic review underscores the uncertainty surrounding the interplay of systemic inflammation, lymphoid organ function, and lymphoma activity in CAR-T-cell therapy. This highlights the complexity of immune responses in these treatments and the need for personalized medicine in cancer treatment. PET scans also show promise in identifying patients receiving CAR-T cell therapy who may face severe risks. The intermediate-term response to CAR-T-cell therapy is consistent with the early metabolic changes seen in both lymphoma lesions and non-targeted lymphoid organs [17]. The search for predictive biomarkers using [^18^F]FDG PET scans is crucial. Identifying reliable markers can enhance treatment planning and patient outcomes. Regarding adverse events, the mean liver and spleen uptake seems to be associated with the occurrence of grade 2 to 4 CRS and ICANS, respectively. As shown by Gui et al. [9], patients with higher SUVmax values before CAR-T-cell infusion are more likely to develop severe CRS and may require preventive treatment. Furthermore, their study found that a high SUVmax before CAR T-cell infusion corresponded to a higher risk and severity of CRS after the infusion. Integrating these biomarkers into the clinical workflow could be useful for early adaptation to patient management; nevertheless, the SUV thresholds found in the study by Marchal and colleagues for the occurrence of CRS grade 2 to 4 (hepatic SUVmean > 2.5) and grade 2–4 ICANS (spleen SUVmean > 1.9) are not unusual in clinical routine. However, these values are not unusual in clinical practice. Given the many existing factors influencing SUV values, they should be handled carefully, particularly in light of the available PET/CT scanner at each institution [15].

Beyond SUVmax and SUVmean, volumetric parameters seem promising in the prediction of toxicity in patients receiving CAR-T-cell therapy. The study by Wang et al. explores the connection between baseline disease burden and side effects, particularly CRS. The lack of a significant correlation between baseline MTV/TLG and overall survival suggests that other factors might play a role in patient outcomes. Nevertheless, it is noteworthy that neurotoxicity was detected in certain patients despite similar TLG and MTV, emphasizing the need for a nuanced understanding of individual responses [19]. The occurrence of CRS in a substantial number of patients is consistent with the existing literature on CAR-T-cell therapy. However, the absence of a clear association with baseline PET parameters raises questions about the complexity of CRS development and the need for further investigation into its predictive factors. An early metabolic response is crucial for remission, emphasizing the need for monitoring treatment progress early on. This aligns with the broader trend in oncology to identify early indicators of treatment success or failure.

Importantly, the study by Morbelli et al. provides valuable insights into hypometabolic patterns associated with CRS and ICANS [16]. The delineation of specific brain regions affected by ICANS offers a detailed understanding of the neurological impact, contributing to the evolving knowledge in this field. In line with the theory that ICANS is primarily a frontal syndrome, patients with this condition show a frontolateral hypometabolic signature. This trend is in line with the frontal lobes’ increased vulnerability to cytokine-induced inflammation [16].

The examination of pre-infusion TGR as a predictor of ICANS and CRS provides a nuanced perspective. The weak correlation with CRS and lack of impact on outpatient planning suggest that TGR alone may not be a decisive factor in treatment management [11].

The findings of our review highlight the capability and reliability of [^18^F]FDG PET imaging in assessing toxicity related to CAR-T-cell treatment. Furthermore, PET imaging provides a sensitive and trustworthy method for anticipating, identifying, and tracking neurotoxicity. When it comes to identifying and tracking toxicity brought on by CAR T-cell therapy, especially in the brain, [^18^F]FDG PET/CT offers reliable and predictive information. These results underline the necessity for further investigation and acknowledgment of the usefulness of PET/CT in therapeutic settings.

The collected literature highlights the potential of PET/CT results to inform response-adapted treatment strategies following CAR-T-cell therapy, as patients who show early indicators of toxicity or a poor response on PET/CT scans may benefit from individualized treatment options to enhance outcomes [13].

In summary, the current literature emphasizes the role of [^18^F]FDG PET/CT in evaluating and predicting toxicity in patients with lymphoma receiving CAR-T-cell therapy, highlighting the evolving nature of research in CAR-T-cell therapy and the need for detailed investigations into various factors influencing treatment outcomes and side effects. The complex interplay of immune responses and individual patient variability necessitates ongoing research to enhance the effectiveness and safety of this promising therapeutic approach.

## 5. Conclusions

[^18^F]FDG PET/CT appears to be a valuable non-invasive tool for predicting and assessing toxicity in patients receiving CAR-T-cell therapy. Additional studies are warranted to increase the collected evidence in the literature.

## Figures and Tables

**Table 1:** Main characteristics of the 11 studies included in the systematic review.

Authors	Year	Country	Journal	*n*. Patients	Sex	Age (Median; Range) in Years
Gui et al. [9]	2024	China	*Eur. J. Nucl. Med. Mol. Imaging*	38	23 M, 15 F	55 (29–74)
Leithner et al. [10]	2024	USA	*J. Hematol. Oncol.*	180	121 M, 59 F	66
Winkelmann et al. [11]	2024	Germany	*Ann. Hematol.*	62	37 M, 25 F	62
Ababneh et al. [12]	2023	USA	*Hematol. Oncol.*	59	33 M, 23 F	66 (35–90)
Crombie et al. [13]	2023	USA	*Hematologica*	329	218 M, 111 F	61 (19–83)
de Boer et al. [14]	2023	The Netherlands	*Blood Adv.*	18	SLR: 7 M, 4 F; HR: 7 M, 2 F	60.5 (35–73)
Marchal et al. [15]	2023	France	*Eur. J. Nucl. Med. Mol. Imaging*	56	36 M, 20 F	Mean: 60.2 (±11.5)
Morbelli et al. [16]	2023	Italy	*J. Neuroimaging*	21	11 M, 10 F	Mean: 55.8 (±11.8)
Derlin et al. [17]	2021	Germany	*Ann. Nucl. Med.*	10	6 M, 4 F	59 (31–74)
Hong et al. [18]	2021	China	*Front. Oncol.*	41	24, 17 F	2 groups: CR: 44 (25–71); Non-CR: 55 (22–70)
Wang et al. [19]	2019	China	*Biol. Blood Marrow Transplant*	19	12 M, 7 F	43 (22–67)

N.: number; M: male; F: female; SLR: sarcoid-like reaction; HR: histiocytic reaction; CR: complete response.

**Table 2:** QUADAS-2 representation evaluating the quality of the studies included in the systematic review based on the four domains of the risk of bias (patient selection, index test, reference standard, flow and timing) and the three domains of the applicability concerns (patient selection, index test, reference standard) ☺ → Low Risk, ☹ → High Risk, ? → Unclear Risk.

Study	Risk of Bias				Applicability Concerns		
	Patient Selection	Index Test	Reference Standard	Flow and Timing	Patient Selection	Index Test	Reference Standard
Ababneh et al. [12]	☺	☹	☺	☺	☺	☹	☺
Gui et al. [9]	☺	☺	☺	☹	☺	☺	☺
Lethner et al. [10]	☺	☺	☺	☺	☺	☺	☺
Winkelmann et al. [11]	☺	☹	☺	☺	☺	☹	☺
Crombie et al. [13]	☺	?	☺	☺	☹	☹	☹
de Boer et al. [14]	☹	☹	☺	☹	☹	☹	☺
Marchal et al. [15]	☺	☹	☺	☹	☹	☺	☺
Morbelli et al. [16]	☺	☺	☺	☺	☺	☺	☺
Derlin et al. [17]	☺	☺	☺	☺	☺	☺	☺
Hong et al. [18]	☺	☹	☺	?	☺	☹	☺
Wang et al. [19]	☹	☺	☺	☺	☹	☺	☺

**Table 3:** List of the 11 studies included in the systematic review with corresponding types of toxicity and main PET findings.

Study	Type of Toxicity	Main PET Findings
Ababneh et al. [12]	CRS, ICANS	CRS was linked with high pre-CAR-T TLG. ICANS was linked with high pre-CAR-T MTV. Elevated pre-CAR-T SUVmax was linked to neurological episodes of grade 3–4.
Crombie et al. [13]	CRS	Elevated baseline lactate dehydrogenase levels, the presence of grade 3 or higher cytokine release syndrome, and a Deauville score of 4 or 5 on the 1-month PET scan were all associated with an increased risk of disease progression, according to a univariable Cox regression analysis.
De Boer et al. [14]	SLR, HR	SLR: symmetric bilateral hilar and mediastinal lymphadenopathy, as well as lymphadenopathy in other areas, accompanied by increased [^18^F]FDG uptake. In the biopsy, there was no sign of lymphoma and only noncaseating epithelioid cell granulomatous inflammation. HR: increased [^18^F]FDG uptake at the site of the initial tumor shortly after CAR-T injection (about one month). A biopsy revealed necrotic lymphoma cells lacking granulomatous processes surrounded by sheets of foamy histiocytic cells.
Derlin et al. [14]	CRS, neurotoxicity	Four patients had CRS and four developed neurotoxicity. Neurotoxicity was linked to higher baseline SUVmax. A decrease in metabolic activity in lymphoid organs was associated with less favorable results, but an early metabolic response was required for remission.
Gui et al. [9]	CRS	Strong direct correlation between pre-infusion SUVmax and the grade of CRS. Moderate direct correlation between pre-infusion TLG and the CRS grade. Pre-infusion SUVmax and CRS risk: higher pre-infusion SUVmax values were linked to an increased risk of developing a higher grade of CRS.
Hong et al. [18]	CRS, coagulation abnormalities (elevated D-dimer levels and prolonged clotting times)	CRS incidence, cytokine levels were considerably higher in patients with higher PET/CT parameters at baseline. Increased D-dimer levels and longer clotting times, two coagulation disorders that might result in bleeding issues, are connected with greater baseline PET/CT parameters.
Leithner et al. [10]	CRS	Grade ≥ 2 CRS was correlated with pre-infusion MTV (odds ratio [OR] for a 100 mL increase: 1.08 [95% confidence interval (CI), 1.01–1.20], *p* = 0.031).
Marchal et al. [15]	CRS, ICANS	Overall survival and progression-free survival were independently predicted by sDmax and TMTV, respectively. Grades 2 through 4 ICANS were associated with greater spleen SUVmean levels, while grades 2 through 4 CRS were linked to higher levels of C-reactive protein and liver SUVmean.
Morbelli et al. [16]	CRS, ICANS	Five of the eleven patients who had CRS went on to develop ICANS. Whereas ICANS was associated with a more widespread hypometabolic pattern in the frontal cortex, CRS without ICANS revealed hypometabolism in bilateral medial and lateral temporal lobes, posterior parietal lobes, and other regions.
Wang et al. [19]	CRS, pseudoprogression	Lower MTV and TLG were associated with mild to moderate CRS, whereas greater MTV and TLG were linked to severe CRS. There appears to be a connection between a higher baseline disease burden and more severe CRS, as evidenced by pseudoprogression and local immune activation.
Winkelmann et al. [11]	CRS, ICANS	The calculated pre-infusion TGR had minimal relationships with the severity of CRS and ICANS, indicating that it might not have a substantial effect on treatment planning or outcome prediction.

CRS: cytokine release syndrome; SLR: sarcoid-like reaction; HR: histiocytic reaction; SUVmax: maximum standardized uptake value; ICANS: immune effector cell-associated neurotoxicity syndrome; sDmax: distance between the two farthest lesions on the body surface, measured and standardized for accuracy; TMTV: total metabolic tumor volume; SUVmean: mean standardized uptake value; MTV: metabolic tumor volume; TLG: total lesion glycolysis; TGR: tumor growth rate; EEG: electroencephalogram.

## Data Availability

Data can be provided upon reasonable request bona fide.
